# Extracranial non-vestibular head and neck schwannomas: a case series with the review of literature

**DOI:** 10.1016/j.bjorl.2021.05.013

**Published:** 2021-06-09

**Authors:** Deviprasad Dosemane, Sushmitha Kabekkodu, Bhagyashree Jaipuria, Suja Sreedharan, Vijendra Shenoy

**Affiliations:** a Kasturba Medical College Mangalore, Manipal Academy of Higher Education, Department of Otorhinolaryngology & Head and Neck Surgery, Karnataka, India; b Mumbai, Maharashtra, India

**Keywords:** Schwannoma, Neurilemmoma, Vagus nerve, Myelin sheath, Histopathology

## Abstract

**Introduction:**

Schwannomas are tumous that arise from Schwann cells. Schwannoma is one of the differential diagnosis for lateral neck swelling.

**Objective:**

In this study, we aim to describe the incidence, presenting clinical features and management of extracranial, non-vestibular schwannomas of head and neck region, along with the review of the literature.

**Methods:**

Patients treated at our tertiary care hospital for head and neck schwannomas for the past 15 years were included in the study. A review of literature on the extracranial head and neck schwannoma was also done.

**Results:**

Twenty-five cases were assessed in this study. Nineteen cases presented as a neck swelling during the initial evaluation. Vagus nerve was the most common nerve of origin, followed by the cervical sympathetic plexus. A rare presentation arising from brachial plexus C5 nerve root was also encountered. A few rare cases of schwannomas arose from the nasal cavity, paranasal sinuses, and oral cavity. Surgical excision was done in all the cases with histopathology suggestive of schwannoma. The nerve of origin of the tumor was identified in nineteen patients. Among them, 11 (58%) were from the vagus nerve, 7 (37%) from the cervical sympathetic chain, and 1 (4%) from the brachial plexus C5 nerve root.

**Conclusion:**

A long-standing unilateral neck mass is the most common presenting complaint in head and neck schwannoma. The diagnosis is mainly based on clinical features and investigations such as imaging. The mainstay of treatment is complete surgical excision. The diagnosis is confirmed on the histopathological study after excision of the lesion. Due to the proximity of the tumor with the involved nerve, palsy may occur. Hence, an accurate preoperative diagnosis of schwannoma is essential.

## Highlights

• Head and neck schwannomas commonly present as a painless swelling in the neck, mostly in middle-aged females.
• Contrast computed tomography and magnetic resonance images can guide the diagnosis as well as surgery.
• Fine needle aspiration cytology with cellular smears is a cost-effective diagnostic tool for superficial lesions.
• Complete enucleation with preservation of nerve of origin is the preferred treatment modality, with negligible recurrence.
• Postoperative nerve palsy may be seen if the nerve of origin is not adequately identified or inaccessible.

## Introduction

Schwannomas are tumors that are of Schwann cell origin, of central, peripheral, or autonomic nervous system.^1^ Verocay coined the term in 1908.^2^ Schwannomas do not arise from the optic and olfactory nerve as they lack those cells. Perineural fibroblast tumour, nerve sheath tumors, neurilemmomas are its other nomenclatures.^1^ Around 25%–45% of these tumors are present in the head and neck region. Schwannoma presents in the 4th to 6th decade of life with no sex predilection.^3^ They are usually seen in the scalp, face, orbit, intracranial cavity, oral and nasal cavity, parapharyngeal areas, mastoid, and larynx. In the head and neck region, the most common occurrence is in the parapharyngeal space. Clinical presentation varies depending on its anatomic site.^3^, ^4^, ^5^, ^6^ A slow-growing swelling and lesion without pain and neurological features are the common presenting symptoms in schwannoma. In the parapharyngeal space, the vagus nerve is the most common nerve of origin (NOO).^7^ Preoperative diagnosis may be aided by radiological imaging like computed tomography (CT) and magnetic resonance imaging (MRI). MRI findings help give an idea about the preoperative estimation of the parent nerve. It also helps to differentiate between different origins of schwannoma.^8^ The usefulness of fine needle aspiration cytology (FNAC) seems to be controversial. After preoperative imaging, complete surgical excision is done as the preferred treatment.^9^ Histopathology shows two main patterns with Antony A and Antony B areas.

There are only a few studies describing extracranial, non-vestibular, head and neck schwannomas and the majority are individual case reports.

This study aims to describe the incidence, clinical features, and management of extracranial, non-vestibular, head and neck schwannomas, with the literature review.

## Patients and methods

The present case series is a retrospective study. Twenty-five cases were included in the study after obtaining the Institutional Scientific and Ethical Committee approval via nº 03-19/85. The inclusion criteria included all the patients diagnosed with extracranial, non-vestibular, head and neck schwannomas, who presented to our department in the past 15 years (March 2005–February 2020). Patients who were diagnosed with intracranial schwannoma, vestibular schwannoma and non-head and neck schwannomas were excluded from the study.

The case files of the patients were procured from the medical records department and analysed. The patient’s epidemiology (age, sex), chief complaints, preoperative imaging, cytology, surgery performed, and histopathology were recorded from the case files.

Histopathological reports were analysed after noting clinical history and examination findings. Various consultants who examined the patients conducted the surgery. Multiple pathologists reported the histopathology.

A review of pertinent literature which was available in English was also done. PubMed search was done using key words “extracranial non-vestibular head and neck schwannoma” in October 2020. Four results were obtained during the search, which was reviewed.

## Result

Twenty-five cases were included in the study, among whom thirteen were females, and twelve were males (1:1 ratio) (Table 1). The mean age was 46 years (range 22–74 years) (Table 1). Except for two patients who suffered from neurofibromatosis, all the others had a solitary head and neck schwannoma.

**Table 1:** Epidemiological and clinical features with nerve of origin of the tumour.

Nº	Age/gender	Presenting complaint	Side	Involved nerve
1	47/M	Neck swelling	Left	Vagus
2	42/F	Neck swelling	Left	Vagus
3	37/M	Neck swelling	Left	Vagus
4	45/F	Cheek swelling	Right	–
5	46/F	Neck swelling	Right	Vagus
6	45/F	Neck swelling	Left	Cervical sympathetic chain
7	43/F	Nasal mass, nasal block	Left	–
8	22/F	Neck swelling	Right	Vagus
9	30/M	Neck swelling	Left	Cervical sympathetic chain
10	50/F	Swelling on the tongue, foreign body sensation in the throat		–
11	37/M	Neck swelling	Right	C5 nerve root brachial plexus
12	36/M	Neck swelling	Right	Vagus
13	40/M	Nasal mass, discharge, and block	Left	–
14	48/F	Neck swelling	Left	Vagus
15	40/F	Neck swelling	Left	Vagus
16	65/F	Neck swelling	Right	Cervical sympathetic chain
17	39/M	Neck swelling	Left	Cervical sympathetic chain
18	57/F	Swelling in the throat	Left	Vagus
19	62/M	Foreign body sensation in the throat, dysphagia, swelling in the tongue		–
20	52/M	Neck swelling	Right	Vagus
21	54/F	Neck swelling	Left	Vagus
22	74/M	Neck swelling	Right	Cervical sympathetic chain.
23	39/M	Swelling in the ear	Left	–
24	45/M	Neck swelling	Right	Cervical sympathetic chain.
25	52/F	Neck swelling	Right	Cervical sympathetic chain.

Unilateral neck swelling was seen in 19 (76%) patients (Table 1). Of the nineteen tumors located in the neck region, 10 cases (53%) had a left-sided mass, and 9 cases (47%) had a right-sided mass. Nasal mass (2) (8%), as well as ear mass (1) (4%), were left-sided, whereas cheek swelling (1) (4%) presented on the right side.

All the twenty-five patients underwent preoperative radiological evaluation.

**Figure 1:**
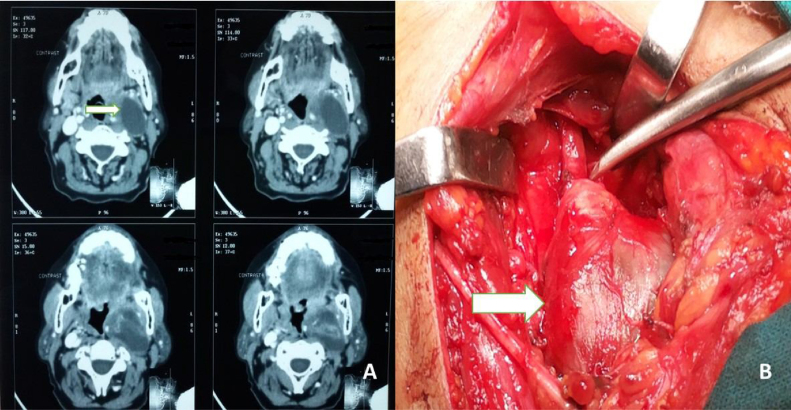
(A) Image showing the axial section of Contrast CT Scan of Vagal Schwannoma. The white arrow pointing towards the right side shows the well-encapsulated schwannoma with cystic change and central enhancing solid component. Vagal schwannoma separates the carotid anteromedially and internal jugular vein anterolaterally. (B) An intraoperative picture of vagal schwannoma is shown by the white arrow pointing towards the right side.

**Figure 2:**
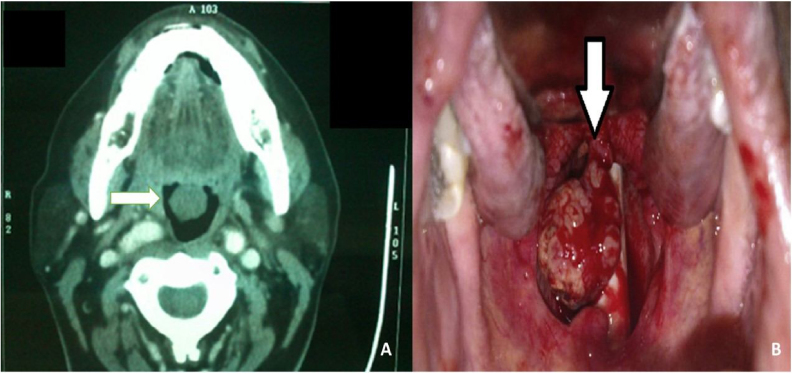
(A) Image showing the axial section of contrast CT scan of Base of Tongue Schwannoma. The white arrow pointing towards the right side shows the schwannoma with stalk attached to the base of the tongue. (B) An intraoperative picture of schwannoma arising from the base of the tongue is shown by the white arrow pointing downwards.

**Figure 3:**
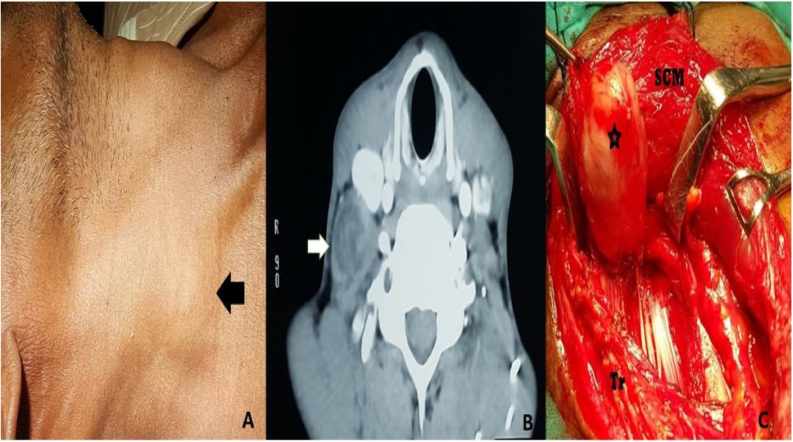
(A) Image showing right-sided brachial plexus schwannoma presenting as swelling in the neck. The lesion is shown by a black arrow pointing towards the left side. (B) Contrast CT scan of brachial plexus schwannoma. A white arrow pointing towards the right side shows the brachial plexus schwannoma, which arises posterolateral to the carotid sheath. (C) Intraoperative picture of brachial plexus schwannoma marked by a black star. Brachial plexus schwannoma is seen deep to sternocleidomastoid. SCM, Sternocleidomastoid; Tr, Trapezius.

**Figure 4:**
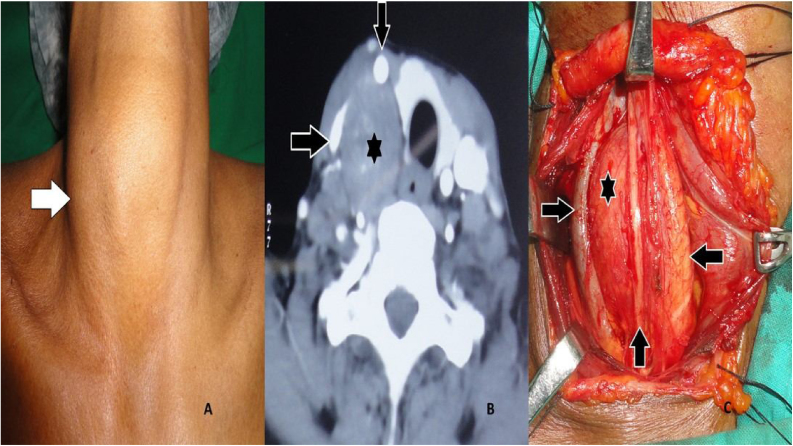
(A) The clinical picture of right-sided cervical sympathetic schwannoma shown by a white arrow pointing towards the right side. (B) Axial section of contrast CT scan, showing the cervical sympathetic plexus schwannoma marked with a black star. A black arrow pointing towards the right side shows the Internal jugular vein compressed and displaced laterally. A black arrow pointing downwards shows the carotid, which is pushed anteriorly by the lesion. (C) A star mark shows the intraoperative picture of schwannoma. The black arrow pointing towards the right side shows the Internal jugular vein compressed and displaced laterally. A black arrow pointing towards the left side shows the carotid, which is pushed anteriorly and medially. The Vagus nerve is seen displaced anteriorly, as shown by the black arrow pointing upwards.

CT with contrast (Figs. 1A, 2A, 3B, 4B) was done in 23 (92%) patients. MRI with contrast was performed in 2 patients (8%). In the present study, only 8 patients (32%) who had easily accessible neck masses underwent preoperative FNAC. It was reported as consistent with a benign nerve sheath tumor in 50% of the cases and was inconclusive in the rest of the cases due to acellular smear.

**Figure 5:**
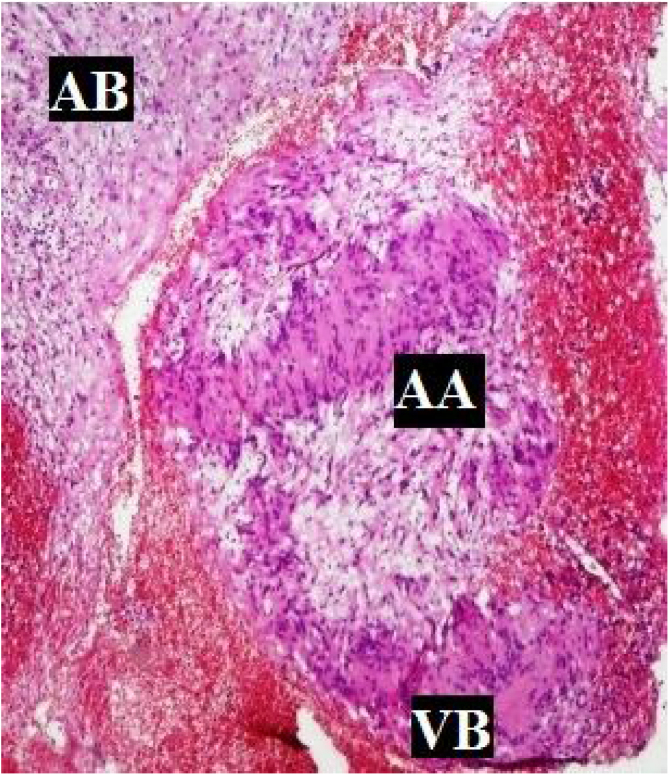
Histological picture showing characteristic features of schwannoma consisting of Antony A and Antony B areas. AA, Antony A areas consist of palisading of the nuclei around a central mass of cytoplasm called Verocay bodies. AB, Antony B areas contain a loose stroma with no distinct pattern by the fibres and cells. VB, Verocay Bodies – the central mass of cytoplasm around which nuclei are seen palisading in Antony A areas.

Complete excision of the tumor with an attempt to identify and preserve the NOO was carried out in all the cases (100%) (Figs. 1B, 3C, 4C). Characteristic Antoni A or B cells (Fig. 5) in histopathology confirmed the diagnosis in all the cases. Immunohistochemistry (IHC) with S-100 was also performed in 2 cases (8%) to confirm the diagnosis.

In this study, the NOO of the tumor (Table 1) was identified in nineteen patients. Among them, 11 (58%) were from the vagus nerve, 7 (37%) from the cervical sympathetic chain, and 1 (4%) from the brachial plexus C5 nerve root.

## Discussion and review of literature

### The present case series

Schwannomas can present in a broad age group, as seen in our case series. We had patients as young as 22 years and as old as 74 years presenting with this disease. The mean age of presentation was 46 years. Schwannomas are equally distributed between genders in the present study, with the male to female ratio being 1:1.

The most typical complaint was swelling in the neck with the lesion in the parapharyngeal space (72%). We had two patients who presented with the lesion in the nasal cavity (8%). Two of the patients had a lesion at the base of the tongue also (Fig. 2A and B) (8%). One patient presented with swelling over the cheek (4%), and another patient presented with swelling over the external ear (4%), showing that schwannomas can be seen in rare sites.

Typically, schwannomas occur unilaterally. However, in this study, two patients (8%) had neurofibromatosis and presented with schwannomas in more than one site, one of it being head and neck.

The present study did not have any patient presenting with symptoms of nerve dysfunction. However, a few patients who had a lesion in parapharyngeal space and base of tongue presented with pressure symptoms like dysphagia. We also came across a patient of vagal schwannoma, who presented with dysphagia and an unusual symptom of giddiness on swallowing.

In the present study, MRI with contrast was performed in two patients, both with schwannoma arising from the base of the tongue. It was reported as fusiform hyperintense mass located at the tongue base. CT scan with contrast (Figs. 1A, 2A, 3B, 4B) was performed in most patients. All the schwannomas were capsulated and fusiform in shape. Homogenous as well as heterogenous contrast enhancement was noted. A few of the cases also had cystic component (Fig. 1A). CT or MRI helped in the diagnosis as well as to delineate the local anatomy before the surgery.

Only 32% of the patients with easily accessible neck masses underwent preoperative FNAC in the present study. It was reported as consistent with a benign nerve sheath tumor in 50% of the cases and was inconclusive in the rest of the cases due to acellular smear.

All the cases underwent complete surgical excision under general anesthesia (Figs. 1B, 2B, 3C, 4C). The incision was decided depending on the location of the tumor. Laser-assisted surgical removal was performed in two cases that presented at the tongue base. None of the cases received radiotherapy.

An attempt to find out the nerve of origin was made in all the cases. 44% of the cases had schwannoma from the vagus nerve (Fig. 1A and B). In 28% of the cases, schwannoma arose from the cervical sympathetic chain (Fig. 4A–C). Brachial plexus C5 nerve root was identified as the nerve of origin in one case (Fig. 3A–C). However, in 24% of cases, the nerve of origin could not be identified.

Histopathology of all the 25 cases was consistent with schwannoma displaying encapsulated tumour with spindle cells. Antony A and Antony B areas were noted (Fig. 5). However, in two doubtful cases, IHC with S-100 was done, which was reported to be positive.

Twenty-three of the cases in the present study had no significant intraoperative or immediate postoperative complications. A patient with left-sided vagal schwannoma developed left-sided vocal cord palsy along with hypoglossal nerve palsy in the post-operative period. Another patient with right-sided vagal schwannoma developed right-sided vocal palsy with hypoglossal palsy as well as right marginal mandibular nerve weakness.

### Clinicopathologic characteristics of Schwannomas

Schwannomas are neoplasms derived from nerve sheath.

Around 25%–45% of these tumors are present in the head and neck region. In the head and neck region, the most common occurrence is in the parapharyngeal space.^3^ Only around 54% of head and neck schwannomas arise from the sinonasal tract. In decreasing order, the sinuses affected are ethmoid, maxillary, nasal fossa and sphenoid sinus.^4^, ^5^ Frontal sinus involvement is rare.

Most of the literature state that schwannomas are prevalent between the third and fifth decade of life. However, a study done by Shrikrishna et al. found more prevalence in males (3:1) with the highest incidence in the second decade of life.^10^

Slow growing swelling and lesion without pain and neurological features are the common presenting feature in schwannoma. In around 5% of the cases, they may be multiple and may be seen with neurofibroma.^6^

Most of the schwannomas arise from the vagus nerve. Less commonly, they may arise from the glossopharyngeal nerve and uncommonly from the superior sympathetic chain.^7^

The clinical symptomatology of sinonasal tumors is nonspecific and varied. It is usually associated with signs of chronic nasal obstruction, like anosmia, running nose, epistaxis, and swelling in the face.^4^

Symptoms of obstruction may be seen if the lesion compresses the airway. Nerve dysfunction may also occur due to the pressure effect. Nerve palsy, like Horner’s syndrome, palsy of vocal cord, motor and sensory dysfunction of upper limb may occur if any main nerve is involved.

Schwannomas of vagus origin displace the internal jugular vein laterally and carotid artery medially. The schwannomas, which are sympathetic chain in origin, displace both carotid artery and jugular vein without delineating.^8^

CT scans with contrast show a well-defined, capsulated, and fusiform lesion with homogeneous contrast enhancement. There may be internal cystic changes becoming more evident as the tumor enlarges.^7^ Liu et al., in a study, stated that the specificity of CT scan was 38%.^11^

Shoss et al., has recommended high-resolution CT to identify the size and extent of the tumor. It also helps demonstrate the degree of vascularity of the tumor and differentiate between malignant and benign conditions.^12^ A retrospective review of radiographic cross-sectional images of parapharyngeal schwannoma found that schwannomas arising from cervical sympathetic chains displace both carotid and jugular vessels without separating them. The study also revealed that carotid and internal jugular vessel were separated in the case of vagal schwannoma.^13^

Preoperative diagnosis of schwannoma is difficult. The various differential diagnoses to be kept in mind are paragangliomas, soft tissue neoplasms like lipoma, fibroma, carotid artery aneurysm, cleft cyst of branchial origin, malignant lymphoma, reactive or metastatic neck lymphadenopathy, and leiomyoma.^9^ Paragangliomas are known for their avid enhancement and high vascularity, which may be associated with erosions on radiology.^14^ Paragangliomas may also show a “salt and pepper” appearance on MRI.^15^ Schwannomas may be differentiated from an aneurysm which shows vascular flow void on imaging. Malignant conditions may show lytic lesions and invasion, which is not seen in schwannoma.^14^

The usefulness of FNAC seems to be controversial. Its accuracy depends on the quality of the specimen and the experience of the pathologist. Liu et al., stated that FNAC had a specificity of around 20%.^11^ Sharma et al., found that 58.3% of FNAC was suggestive of benign nerve sheath tumor (BNST), and only 8.3% of the tumors showed characteristic features suggestive of schwannoma. The sensitivity of FNAC in diagnosing BNST was 71.4% and hence can be used as a minimally invasive, cost-effective option in the diagnosis.^15^ Cytological features of schwannoma were also explained in a study by Sun Young Kim et al. This study stated that the cytological features common to all cases of schwannoma usually corresponded to Antony A type of tissue consisting of fragments of cohesive fascicles with variable cellularity, a dense fibrillary substance with palisading nuclei and Verocay bodies. Antony B areas consisted of a short spindle or scattered wavy cells in the myxoid background with microcyst formations.^16^

The preferred treatment is complete excision by surgery. During the surgery, schwannomas appear as whitish-yellow, circumscribed lesions. Dissection of mass from the nerve of origin taking care of neurological pathway should be done.^9^ Based on the clinical diagnosis, site and dimensions of the mass and surgeon’s choice, the surgical approach may vary. Intracapsular enucleation is a commonly followed surgical technique, with a success rate between 30%–86%.^17^, ^18^

A study done by Kei Ijichi et al., suggested that using an electromyographic system to stimulate and map the nerve of origin during microsurgery for enucleation helps reduce the post-operative nerve palsy for motor nerve schwannomas. However, since sympathetic and sensory nerves do not get stimulated by EMG, this method cannot be used for sympathetic schwannoma.^19^ Schwannomas are not radiosensitive, and so radiotherapy has no role in the treatment.^20^

Histopathology shows two main patterns- Antoni A and Antoni B. Palisading of the nuclei around a central mass of cytoplasm called Verocay bodies is seen in Antony A areas. Antony B areas contain a loose stroma with no distinct pattern by the fibres and cells. A mixture of both features can also be there. Other features like hemorrhage, necrosis and cystic degeneration may also be seen.^21^ IHC with S-100 is positive in most schwannomas, which is evident with diffuse cytoplasmic staining. Hence in doubtful cases, IHC with S-100 can be done to confirm the diagnosis.

A study done in Japan on functional nerve preservation in schwannomas found that motor schwannoma enucleation with intraoperative EMG guidance had no complications post-surgery, whereas, in sympathetic schwannomas, chances of postoperative neurological dysfunction like Horner syndrome were seen.^19^ Intracapsular enucleation preserved nerve function by more than 30%, as compared with complete tumor resection.^17^ A study had stated that the postoperative nerve palsy rates were 100% with resection and division of nerve of origin, 67% with intracapsular enucleation and 50% with debulk operations.^11^

The post-surgical recurrence of schwannoma is almost nil.^5^, ^10^, ^11^, ^19^ However, a study by Liu et al., stated that followup MRI of a patient who underwent a debulk operation showed a slow progression of the tumor at 2 years.^11^ The patient’s followup was noted for six months in the study, and no evidence of recurrence on clinical examination occurred.

Transformation into malignancy in solitary tumors is rare.^9^

### Literature review

A 10-year retrospective study on 46 patients conducted in Shanghai, China, by Baoxin Wang et al., (Table 2) revealed that all the tumors examined were benign. Females were seen to be more affected than males. 52% of the cases presented as an asymptomatic mass with a mean size of 4.5 cm. The brachial plexus was the most common nerve of origin in the study.^22^

**Table 2:** Table representing the epidemiological data along with the chief complaints, investigations, nerve of origin, treatment, complications, and the follow-up data of the reviewed literature on extracranial non-vestibular head and neck schwannomas.

Nº	Author	Mean Age/n	Sex	Chief complaints	Investigations	Nerve of origin	Treatment	Post-op complications	Follow up
1	Baoxin Wang et al.,	47.6/46	F (56.5%)	Asymptomatic neck mass (61%), neurological symptoms (30%), obstructive symptoms (9%)	CT (65.2%) MRI (41.3%) USG (50%) FNAC (13.04%)	Brachial plexus (28.3%), Vagus (8.7%), Sympathetic chain (4.4%), Facial nerve (4.4%), Cervical plexus (4.4%), Hypoglossal nerve (2.2%), Unidentified (47.8%).	Complete surgical resection	Neurological sequelae (43.5%)	12–96 months – No recurrence
2	Gavin et al.,	48/21	F (67%)	Unilateral neck mass (76%), Others-neurological, reflux symptoms etc.	CT/MRI (71%)	Vagus (28.6%), Cervical sympathetic chain (23.8%), Facial nerve (19%), Brachial plexus (9.5%), Trigeminal nerve (4.8%), Accessory nerve (4.8%), Hypoglossal nerve (4.8%).	Complete surgical resection	Neurological deficit (76%)	1–108 months – No recurrence
					USG (4.7%)				
					FNAC (42.86%)				
3	Bondi et al,	42.1/18	F (61.1%	Painless neck mass (61.1%), Others-dysphonia, dysarthria etc.	CT/MRI (77%)	Vagus (38.9%), Hypoglossal (11.11%), Lingual (5.5%), Spinal accessory (5.5%), Cervical plexus (5.5%), Brachial plexus (5.5%), Others-tongue, lip, nose and neck.	Radical surgery (94.4%), Partial excision (1 case)	Neurological deficit (11.1%)	6–120 months – No Recurrence
					USG with FNAC (50%)				
4	Lahoti et al.,	38/8	F (72%)	Neck mass (100%)	CT/MRI (100%)	Vagus (50%), Cervical sympathetic chain (25%), Accessory nerve (12.5%).	Complete surgical resection	Neurological deficit (50%)	24 months – No recurrence
					FNAC (75%)				

n, Number of patients included in the study; F, Females.

In a retrospective study (Table 2) by Gavin et al., the mean age at diagnosis was 48 years with a female predominance. 76% presented with complaints of unilateral neck swelling. Vagus and cervical sympathetic chains were the most common nerve of origin in this study. No recurrence was noted in 2 years of follow-up.^23^

A retrospective study by Bondi et al., (Table 2) which included 18 patients, revealed that painless neck mass was the most common presenting complaint in patients with head and neck schwannoma. Female predominance was noted with a mean age of 42.1 years. Half of the group underwent ultrasonography with fine-needle aspiration, which was diagnostic on 30% of the cases. MRI was diagnostic in 77% of the cases. No recurrence was noted on follow up period of 6–120 months.^24^

Lahoti et al., in a 2-year retrospective study (Table 2), found female predominance with a mean age at diagnosis being 38 years. Vagus and cervical sympathetic chain were the most common nerve of origin. Followup at 24 months revealed no signs of recurrence after a complete surgical excision.^20^

The main limitation of this study is that it is a retrospective study, involving patients managed by multiple consultants. A prospective study may be done on this topic with a single assessor, with adequate follow up of the patients.

## Conclusion

Schwannomas can be considered one of the differentials of lateral neck swelling and are more common in middle-aged females. They can also present at other areas of the head and neck, though rare. Preoperative CT or MRI scan with contrast can guide in diagnosis as well as surgical excision, avoiding any iatrogenic damage. FNAC can be considered as a cost-effective diagnostic tool, provided the lesion is accessible, and cellular smears are obtained. Complete surgical enucleation with preservation of nerve of origin is the preferred treatment modality. Depending on the nerve of origin, the patient should be counselled regarding the possibility of postoperative neural deficit. Following surgical excision, the chances of recurrences are rare.

## Funding

This research did not receive any specific grant from funding agencies in the public, commercial or not-for-profit sectors.

## Ethics approval

This retrospective study was conducted after obtaining Institutional Ethics Committee Approval (Manipal Academy of Higher Education- Kasturba Medical College, Mangalore), via No. IEC KMC MLR 03-19/85.

## Conflicts of interest

The authors declare no conflicts of interest.
